# Comparison between public and private sectors of care and disparities in adverse neonatal outcomes following emergency intrapartum cesarean at term – A retrospective cohort study

**DOI:** 10.1371/journal.pone.0187040

**Published:** 2017-11-17

**Authors:** Woonji Jang, Christopher Flatley, Ristan M. Greer, Sailesh Kumar

**Affiliations:** 1 Mater Research Institute—University of Queensland, South Brisbane, Queensland, Australia; 2 Faculty of Medicine, The University of Queensland, Brisbane, Australia; University of Texas at Austin, UNITED STATES

## Abstract

**Background:**

Perinatal outcomes may be influenced by a variety of factors including maternal demographics and medical condition as well as socio-economic status. The evidence for disparities in health outcomes stratified by type of care (public or private) is lacking. The aim of this study was to investigate short term neonatal outcomes following category 1 and 2 emergency cesareans at term between publicly and privately funded women at a single major tertiary centre in Australia. Category 1—immediate threat to life (maternal or fetal); Category 2—maternal or fetal compromise that is not immediately life-threatening.

**Methods:**

This was a retrospective, cross sectional study of 61355 term singleton babies born at the Mater Mother’s Hospital in Brisbane, Australia in 2007–2014. We collected data from the hospital’s maternity database and compared maternal demographics, indications for cesarean and neonatal outcomes for publicly and privately funded women.

**Results:**

Over the study period there were 32477 public and 28878 private, term singleton births. Compared to the publicly funded cohort, privately insured women were older, had lower BMI, were of Caucasian ethnicity, Australian born, nulliparous, had shorter labors and had lower rates of hypertensive disorders and diabetes. The most common indications for category 1 and category 2 cesareans in combination were non-reassuring fetal status followed by failure to progress in labor and malpresentation. For both category 1 and 2 cesareans, neonatal outcomes (Apgar score <7 at 5 minutes, abnormal cord gases, Neonatal Critical Care Unit admission rates, rates of severe respiratory distress and jaundice) were significantly worse in the publicly funded compared to the privately insured cohort Multivariate analyses controlling for maternal age, ethnicity, country of birth, parity, hypertension, diabetes mellitus, gestational age at birth and length of labour confirmed that private insurance status was highly protective for the perinatal outcomes of Apgar score <7 at 5 minutes (aOR 0.26, 95% CI 0.13–0.55), admission to NCCU (OR 0.51, 95% CI 0.30–0.92) and respiratory distress (aOR 0.60, 95% CI 0.41–0.86).

**Conclusion:**

Birth in the private health sector was inversely associated with adverse neonatal outcomes following category 1 and 2 cesareans.

## Introduction

Globally there are marked differences in cesarean, induction of labour, operative vaginal delivery and episiotomy rates in North America[1], Australasia[2] [3] [4], Europe[5] [6] [7], the Middle East[8] and Latin America[9]. It is likely that there are many reasons for these disparities in obstetric interventions, including maternal request[10] [11] [12] and fear of litigation[13]. In Australia, private health insurance rates have gradually increased with almost 47% of the population now holding hospital coverage. Furthermore, for the demographic cohort of 15–50 years of age, more women than men hold private health insurance with high rates of utilization of health coverage for private maternity care. Obstetric intervention rates in Australia differ widely between the public and private sector with the highest rates seen in private hospitals, intermediate rates for privately insured women birthing in public hospitals and lowest rates for women without private health insurance[14].

A recent large study utilizing data from the Australian perinatal data collection[15] suggested that although intervention rates were higher in privately insured women, overall neonatal outcomes were significantly better in this cohort. Another study from New York showed that neonatal outcomes were significantly better both in privately insured women as well as women without private health insurance but birthing in a private setting[1]. There is however a paucity of neonatal outcome data relating to the urgency of emergency intrapartum cesarean at term between publicly funded and privately insured women. Classification of the degree of urgency of cesarean is generally based on one of four categories[16]: Category 1—immediate threat to life (maternal or fetal); Category 2—maternal or fetal compromise that is not immediately life-threatening; Category 3—needing early delivery but no maternal or fetal compromise; or Category 4—delivery at the convenience of the patient or obstetric team. Professional bodies such as the American Congress of Obstetrics and Gynaecology[17], National Institute of Clinical Excellence in the United Kingdom[18] and the Royal Australian and New Zealand College of Obstetricians and Gynaecologists[19] all have broadly similar recommendations in that the decision to delivery interval for Category 1 cesarean should be no longer than 30 minutes.

The objectives of this study were to firstly compare the demographics, major co-morbidities and modes of delivery in non-insured (“publicly funded”) and privately insured women (“privately funded”) with singleton, term deliveries at a single major tertiary centre in Australia and secondly to compare neonatal outcomes following category 1 and 2 cesareans at term between these two groups.

## Materials and methods

### Study setting

This was a retrospective cross sectional study of all women with singleton pregnancies who underwent either a Category 1 or Category 2 cesarean at term (37–42 weeks), at the Mater Mothers’ Hospital in Brisbane, Australia between January 2007 and October 2014. The Mater Mother’s Hospital is the largest maternity hospital in Australia with a combined public-private birth rate of almost 10,000 per annum. This equates to 1 in 6 births in the state of Queensland. All women, regardless of health insurance status, birth in one delivery suite staffed by a common team of midwives who care for women in conjunction with both the public obstetric team and individual private obstetricians. Intrapartum care for all women is guided by hospital guidelines and policies to which all obstetric staff (public and private) have to adhere. When privately funded women require either an operative vaginal delivery or emergency cesarean, a specialist pediatrician is normally in attendance for the birth. The birth itself is conducted by the private specialist obstetrician. In contrast, public women are cared for by two on duty obstetricians in specialist training supported by a specialist obstetrician who is able to attend within 20–30 minutes if required. The on duty trainee obstetrician is credentialed to perform term cesareans without supervision as well as instrumental vaginal deliveries. A trainee neonatologist attends all operative vaginal births and emergency cesareans for publicly funded women. A specialist public neonatologist may either be on site or in close proximity and able to attend rapidly if required.

Category 1 and 2 cesareans are the two most urgent categories of cesareans and are usually performed for urgent fetal or maternal indications as described earlier. Categorization of the urgency of the caesarean section was made contemporaneously by a member of the obstetric team and the primary indication for operative birth was used for analysis.

Maternal demographic data and perinatal outcome data were collected from the hospital’s maternity database and cross-referenced with the maternal and fetal medicine and neonatal databases to ensure robust data ascertainment for maternal demographics, gestation, mode of delivery and neonatal outcomes.

### Inclusion and exclusion criteria

Inclusion criteria were singleton pregnancy, gestation ≥37 weeks, absence of major congenital malformation and absence of any known fetal demise at any gestation prior to labour. We excluded from analysis multiple pregnancies, pre-term gestation (<37 weeks), major congenital malformation and known fetal demise at any gestation prior to labor.

### Maternal and perinatal variables

Maternal demographic information included age, body mass index (BMI), ethnicity, country of birth, maternal medical conditions (hypertension/pre-eclampsia and diabetes) and patient admission status (public or private). Hospital sector of birth (public or private) was determined at the time of booking and updated if subsequently changed. In general, public patients do not have private health insurance and private patients have private health insurance, although this is not invariably the case. Although some patients may not have private health insurance they may still elect to be cared for a private obstetrician. For the purposes of this study, these patients would fall under the “private” category. Mode of delivery was classified as normal vaginal delivery (NVD), instrumental delivery (forceps or vacuum), or cesarean (planned cesarean and category of emergency cesarean).

Perinatal outcome data was collected for women who experienced a Category 1 or Category 2 cesarean. Data included gestation at delivery to the nearest week, birth weight, Apgar score at 5 minutes, umbilical arterial pH and lactate, neonatal complications defined by attending neonatologist (e.g. respiratory distress, seizure, jaundice and infection), neonatal critical care unit (NCCU) admission, and neonatal death prior to discharge. Data on the category or urgency and indication for cesarean (non-reassuring fetal status, failure to progress in labor, malpresentation, failed instrumental delivery, antepartum hemorrhage/placenta previa, cord prolapse, uterine rupture, intrapartum hemorrhage and maternal disease) were also collected.

### Ethical approval

Research Ethics and Governance approvals for this study were granted by the Mater Human Research Ethics and Governance Committee (HREC/13/MHS/104).

### Statistical analysis

Data integrity was assessed using a year by year analysis to identify inconsistencies of reporting between years. Where data integrity was questionable with sudden drops in outcomes that could not be accounted for by change in policy or treatment, those variables were excluded from any analysis. Efforts were made to correct missing and data entry errors through searches of individual patient records. Where data were collected with different degree of outcomes between years, these variables were collapsed into dichotomous variables to indicate whether the outcome occurred or not. Where only the outcomes were recorded, after discussion with data custodians it was determined that it was reasonable to assume that missing data indicated that the outcome had not occurred.

Normally distributed variables were compared using a two sample t-test or Analysis of Variance (ANOVA) if there were three or more groups. Non-normally distributed variables were compared using a Wilcoxon Rank Sum test for two groups or Kruskall-Wallis test if there were three or more groups. Frequencies were compared using a chi-square test. The proportion of infants in each category of indication for cesarean section (CS indication) was compared using a z-test for two proportions. Summary statistics are reported as mean (SD) or median (IQR) as appropriate. A probability (p) value of <0.05 was used to define statistical significance. Multivariate logistic regression analysis to assess the influence of maternal insurance status on neonatal outcomes for women who required a category 1 or category 2 cesarean was also performed. The results are reported as adjusted Odds Ratios (aOR) and 95% Confidence Intervals (95% CI). Statistical analysis for this study was performed using Stata 13 (StataCorp. Stata Statistical Software: Release 13. College Station, TX: StataCorp LP).

## Results

Table 1 details the demographic characteristics of the study population. Over the study period a total of 61,355 women met the inclusion criteria. Of these, 52.9% (32,477/61,355) were recorded as publicly funded patients and 47.1% (28,878/61,355) were privately insured. Compared to the public cohort, privately insured women were more likely to be older, have lower BMI, have Caucasian ethnicity and be Australian born. The proportion of nulliparous women was 2% higher in the private cohort. Overall, publicly funded women had higher rates of hypertensive disorders and diabetes. The median time in labour was 35 minutes longer in publicly funded women than in privately insured women.

**Table 1:** Maternal demographics of singleton term deliveries in publicly and privately funded women.

	Public (n, %) n = 32,477 (52.9)	Private (n, %) n = 28,878 (47.1)	p value^#^
**Age**^	29.7(5.6)	33.4(4.2)	<0.001
BMI*	23.05 (20.32–26.99),	22.53 (20.48–25.46)	<0.001
**Ethnicity**			
**Caucasian**	18,049 (55.6)	24,227 (83.9)	<0.001
**Indigenous (ATSI)**	950 (2.9)	37 (0.1)	<0.001
**Asian**	5192 (16.0)	1658 (5.7)	<0.001
**Other**	8286 (25.5)	2956 (10.2)	<0.001
**Born outside of Australia**	15,108/32,469 (46.5)	6606/28,877 (22.9)	<0.001
**Parity**			
**P0**	14,774/32,471 (45.5)	13,715/28,874 (47.7)	<0.001
**≥P1**	17,697/32,471 (54.5)	15,159/28,874 (52.7)	<0.001
**Hypertension (including PET)**	1843/32,412 (5.7)	1418/28,850 (4.9)	<0.001
**Diabetes**	1029/32,298 (3.2)	815/28,721 (2.8)	<0.001
Total length of labour in minutes*	350/24,620 (203–548)	315/16,495 (194–480)	<0.001

BMI: body mass index, PET: Pre-eclampsia, ATSI: Aboriginal and Torres Straits Islander.

Data presented as Number (percentage) unless otherwise specified.

^#^Chi-square.

*Data presented as Median (interquartile range).

^ Data presented as Mean (standard deviation).

Table 2 details the modes of birth and the cesarean categories for both cohorts. There were more normal vaginal deliveries, forceps deliveries and emergency caesarean births in the publicly funded compared to the privately insured cohort. Category 1 and 2 emergency CS were also more common amongst publicly funded women. The overall emergency caesarean birth rate was 2.7% higher in publicly funded women than in privately insured women. Planned caesarean births occurred more commonly among privately insured women.

**Table 2:** Modes of delivery of singleton term births in publicly and privately funded women.

	Public (n, %) 32,477 (52.9)	Private (n, %) 28,878 (47.1)	p value^#^
**Normal Vaginal Delivery**	19,636 (60.5)	11,986 (41.5)	<0.001
**Instrumental**	4240 (13.1)	3961(13.7)	0.02
**Forceps**	1033 (3.2)	660 (2.3)	<0.001
**Vacuum**	3207 (9.9)	3301 (11.4)	<0.001
**Cesarean**	8597 (26.5)	12,930 (44.8)	<0.001
**Planned CS**	3673 (11.3)	9318 (32.3)	<0.001
**Emergency CS**	4924 (15.2)	3612 (12.5)	<0.001
**Category 1 CS**	1232 (3.8)	529 (1.8)	<0.001
**Category 2 CS**	2377 (7.3)	1845 (6.4)	<0.001

CS: Cesarean Section.

Data presented as Number (percentage) unless otherwise specified.

^#^Chi-square.

Table 3 details the demographic characteristics of the publicly funded and privately insured cohorts stratified by category of cesarean section. In the Category 1 and 2 groups, age, BMI, ethnic background, country of birth and prevalence of hypertension, gestational diabetes, heart disease and autoimmune disease were broadly similar to the entire cohort. The median recorded length of labour before a Category 1 cesarean was performed was 42 minutes longer in the publicly funded compared to the privately insured cohort but this difference just failed to attain significance (p = 0.06). The median length of labour before a Category 2 cesarean was performed was significantly longer (157 minutes) in the publicly funded cohort.

**Table 3:** Maternal demographics of singleton term Category 1 & 2 cesareans.

	Category 1 CS, n = 1761			Category 2 CS, n = 4222		
	Public (n = 1232)	Private (n = 529)	p value^#^	Public (n = 2377)	Private (n = 1845)	p value^#^
**Age^**	29.8 (5.7)	33.3 (4.1)	<0.0001	30.0 (5.5)	33.0 (4.1)	<0.001
**BMI**	23.4 (20.7–27.2), n = 1215	22.8 (20.8–26.1), n = 525	0.20	23.6 (21.0–28.0), n = 2344	23.1 (21.0–26.4), n = 1824	0.002
**Ethnicity**						
**Caucasian**	638 (51.8)	450 (85.1)	<0.001	1275/2377 (53.6)	1544/1845 (83.7)	<0.001
**Indigenous (ATSI)**	40 (3.2)	0 (0.0)	<0.001	64/2377 (2.7)	3/1845 (0.2)	<0.001
**Asian**	211 (17.1)	32 (6.0)	<0.001	428/2377 (18.0)	145/1845 (7.9)	<0.001
**Other**	343 (27.8)	47 (8.9)	<0.001	610/2377 (25.7)	153/1845 (8.3)	<0.001
**Born outside of Australia**	635 (51.5)	150 (28.4)	<0.001	1208/2377 (50.8)	480/1845 (26.0)	<0.001
**Hypertension (including PET)**	42 (3.4)	14 (2.60)	0.40	122/2372 (5.1)	75/1844 (4.1)	0.1
**Diabetes**	31/1229 (2.5) 24 (1.8)	10/528 (1.2)	0.42	55/2371 (2.3)	39/1843 (2.1)	0.66
Total length of labour in minutes*	499 (318–690) n = 347	457 (313–595) n = 141	0.06	647 (457–826) n = 561	490 (326–634), n = 351	<0.001

BMI: body mass index, ATSI: Aboriginal and Torres Straits Islanders, CS: Cesarean Section.

Data presented as Number (percentage) unless otherwise specified.

^#^Chi-square.

*Data presented as Median [interquartile range].

^ Data presented as Mean (standard deviation).

Table 4 details the primary indications for category 1 and 2 cesareans. For Category 1 cesareans the two most common indications for both the publicly funded and privately insured cohorts were non-reassuring fetal status and failure to progress in labour. For Category 2 cesareans the leading indication for delivery was failure to progress, followed by non-reassuring fetal status. Overall, for both Category 1 and 2 cesareans **in combination**, almost 40% of procedures were performed for non-reassuring fetal status. Failure to progress in labour, malpresentation and failed instrumental delivery were the next most common indications. Failure to progress was more common in the privately insured cohort.

**Table 4:** Primary indications for Category 1 and Category 2 cesarean.

	Category 1 CS			Category 2 CS		
	Public (n = 1232)	Private (n = 529)	p value^#^	Public (n = 2377)	Private (n = 1845)	p value^#^
**Non-reassuring fetal status**	831 (67.4)	331 (62.6)	0.06	559 (23.5)	581 (31.5)	<0.001
**Failure to progress**	123 (9.9)	59 (11.5)	0.51	1184 (49.8)	738 (40.0)	<0.001
**Malpresentation**	64 (5.2)	22 (4.2)	0.42	261 (11.0)	141 (7.6)	0.0003
**Failed instrumental**	90 (7.3)	42 (7.9)	0.71	49 (2.1)	37 (2.0)	0.99
**Antepartum hemorrhage**	9 (0.7)	5 (0.9)	0.86	18 (0.8)	21(1.1)	0.26
**Placental abruption**	17 (1.4)	14 (2.6)	0.05	2 (0.08)	5 (0.3)	0.25 †
**Maternal disease**	14 (1.1)	3 (0.6)	0.40	32 (1.3)	52 (2.8)	0.001
**Cord prolapse**	26 (2.1)	18 (3.4)	0.15	5 (0.2)	0 (0.0)	0.07 †
**Chorioamnionitis**	2 (0.2)	2 (0.4)	0.74	6 (0.2)	7 (0.4)	0.65
**Intrapartum hemorrhage**	6 (0.5)	4 (0.8)	0.73	3 (0.1)	2 (0.1)	1.0†
**Other**	50 (4.1)	29 (5.5)	0.23	258 (10.8)	261 (14.1)	0.001

CS: Cesarean Section. **‘Other’ includes women where indication for CS was not recorded.**

^#^Chi-square (unless otherwise stated).

†Fisher's exact test.

Table 5 details the perinatal outcomes by category of caesarean section. For Category 1 cesareans the median gestational age (40 weeks) at birth was similar for both cohorts, however the slight difference in distribution made this difference statistically significant, suggesting that in those delivering over 40 weeks, publicly funded patients delivered slightly later than women with private health insurance. Median birth weight and the proportion of small babies (Birth weight <5^th^ or 10^th^ centile) did not differ between the groups. The proportion of infants with Apgar score <7 at 5 minutes, acidosis, NCCU admission, severe respiratory distress and jaundice were higher in the public group. For Category 2 cesareans the median gestational age at birth was lower in the privately insured cohort. In babies delivered by women in the publicly funded cohort, there was a higher proportion with an Apgar score <7 at 5 minutes, severe respiratory distress, jaundice and admission to NCCU.

**Table 5:** Perinatal outcomes of term Category 1 & 2 cesareans.

	Category 1 CS			Category 2 CS		
	Public N = 1232	Private N = 529	p-value^#^	Public (n = 2377)	Private (n = 1845)	p-value^#^
Gestation at delivery*	40 (39–41)	40 (39–40)	0.0001	40 (39–41)	39 (38–40)	<0.001
**Birth weight (g)**	3420.9 (518.6)	3405.2 (453.9)	0.52	3565.2 (545.9)	3504.3 (474.1)	<0.001
**BW <10**^**th**^ **centile**	136 (11)	53 (10)	0.984	238 (10)	182 (9.9)	0.87
**BW <5**^**th**^ **centile**	68 (5.5)	26 (4.9)	0.944	119 (5)	90 (4.9)	0.85
**Apgar <7 at 5 min**	72/1229 (6.0)	12/529 (2.0)	0.001	59/2377 (2.5)	13/1845 (0.7)	<0.001
**Umbilical arterial pH <7.2/Lactate >4mmol/L**	643/1126 (57.1)	202/342 (59.1)	0.52	745/2026 (36.8)	270/581 (46.5)	<0.001
**NCCU admission**	69 (5.6)	15 (2.8)	0.01	62 (2.6)	15 (0.80)	<0.001
**Severe Respiratory Distress**	141 (11.4)	41 (7.8)	0.005	163 (6.9)	66 (3.6)	<0.001
**Jaundice**	169 (13.7)	39 (7.4)	0.0002	272 (11.4)	124 (6.7)	<0.001
**Infection**	4 (0.3)	1 (0.2)	0.62	6 (0.3)	2 (0.1)	0.28
**Neonatal death**	5 (0.4)	1 (0.2)	0.47	3 (0.1)	0 (0.0)	0.13

NCCU: Neonatal Intensive Care Unit, BW: Birth weight, CS: Cesarean Section.

Data presented as Number (percentage) unless otherwise specified.

^#^Chi-square.

*Data presented as Median (interquartile range).

Table 6 details perinatal outcomes for combined category 1 and 2 cesarean sections. When both Category 1 and 2 CS were combined, overall perinatal outcomes were significantly worse in the publicly funded cohort. The only exception to this was acidosis at birth which was higher in the privately insured cohort. This finding is likely to be skewed because of the higher proportion of acidotic neonates in the Category 2 private cohort.

**Table 6:** Combined Category 1 & 2 perinatal outcomes.

	Public Cat 1 & 2 CS (n = 3609)	Private Cat 1 & 2 CS (n = 2374)	p value^#^
Gestation at delivery*	40 (39–41), n = 3609	39 (38–40), n = 2374	<0.001
**Birth weight (g)** *	3515 (541.1), n = 3609	3482 (471.4), n = 2374	0.01
**BW <10**^**th**^ **centile**	367 (10.2)	234 (9.9)	0.96
**BW <5**^**th**^ **centile**	178 (4.9)	117 (4.9)	0.76
**Apgar <7 at 5 min**	128/3609 (3.5)	21/2374 (0.9)	<0.001
**Umbilical arterial pH <7.2/Lactate >4**	1388/3152 (44.0)	472/923 (51.1)	<0.001
**NCCU admission**	131/3609 (3.6)	30/2374 (1.3)	<0.001
**Severe Respiratory Distress**	304/3099 (9.8)	110/2199 (5.0)	<0.001
**Jaundice**	441/3609 (12.2)	163/2374 (6.9)	<0.001
**Infection**	13/3609 (0.36)	2/2374 (0.08)	0.04
**Neonatal death**	8/3609 (0.22)	1/2374 (0.04)	0.08

NCCU: Neonatal Intensive Care Unit, BW: Birth weight, CS: Cesarean Section.

Data presented as Number (percentage) unless otherwise specified.

^#^Chi-square.

*Data presented as Median (interquartile range).

Multivariate analyses controlling for maternal age, ethnicity, country of birth, parity, hypertension, diabetes mellitus, gestational age at birth and length of labour confirmed that private insurance status was significantly protective only for the perinatal outcomes of Apgar score <7 at 5 minutes (aOR 0.26, 95% CI 0.13–0.55), admission to NCCU (OR 0.51, 95% CI 0.30–0.92) and respiratory distress (aOR 0.60, 95% CI 0.41–0.86) for category 1 and 2 cesareans.

## Discussion

In this Australian cohort, publicly funded patients were younger, had higher BMI, and higher rates of non-Caucasian ethnicity, being born overseas, hypertension and diabetes than privately insured women. Publicly funded women also had higher rates of normal vaginal deliveries and correspondingly lower rates of instrumental and cesarean births. Regardless of category of emergency cesarean, our results suggest that neonatal outcomes for infants born to women in the publicly funded cohort were poorer overall compared to those with private health insurance, although there appeared to be some independent contribution to outcomes from birthweight, gestational age at delivery and ethnicity.

The reasons for the disparities in perinatal outcomes are not immediately clear from the data available for this study. Although it is known that perinatal outcomes tend to be worse in women who have significant medical co-morbidities [20], in our study there was no difference in the prevalence of diabetes or hypertension in the combined category 1 and category 2 groups. We also deliberately confined our analyses to term pregnancies as this would have negated the potentially confounding effect of prematurity which is known to greatly influence perinatal outcomes. The incidence of birthweights either below the 5^th^ or 10^th^ centile for gestation was similar in both groups thereby reducing the likelihood that fetal growth restriction was responsible for the poorer outcomes in the public cohort. Aside from maternal and perinatal factors the other differences between the groups were the seniority of obstetric and pediatric staff involved in the immediate care of the two cohorts. Privately insured women were cared for by a specialist obstetrician with a specialist pediatrician in attendance at the time of an emergency cesarean.

Operative intervention was also more likely to take place earlier in labour in privately insured women compared to their publicly funded counterparts. Babies from women who were publicly funded were clearly exposed to the stress of uterine contractions for a longer period and may therefore be born in a poorer condition compared to babies in the privately insured group. However, time in labour did not influence Apgar score <7 at 5 minutes or admission to NCCU. It is possible that the shorter duration of labour in the privately insured group combined with perhaps more effective neonatal resuscitation by a more experienced pediatrician may explain the better neonatal outcomes in this group. It is also entirely possible that private obstetricians are more cautious and therefore tend to intervene sooner than their public colleagues. There is evidence[1] that privately insured patients are perceived as posing a greater medical-legal risk to obstetricians thereby resulting in greater intervention rates which may in turn result in better neonatal outcomes.

In some North American studies the absence of private health insurance coverage appears to confer a greater risk of adverse outcomes with critically ill uninsured patients experiencing higher hospital mortality than insured patients[21] [22] suggesting that lack of insurance may be an independent risk factor for death. In a study from Argentina[23], uninsured obstetric patients were noted to be more severely ill on admission and experienced worse outcomes than insured patients. Other studies have shown that for women admitted to the intensive care unit, non-obstetric causes of admission were more frequent among uninsured than insured women which may suggest a higher prevalence of co-morbidities[24] and more severe disease[25] [20]. Although our study was confined to neonatal outcomes following the two most urgent categories of cesarean, demographic analyses of our data did not demonstrate any difference in maternal co-morbidities (diabetes or hypertension) that could explain the discrepancy in neonatal outcomes in our cohort.

In a large study from Ireland[5] of more than 400,000 births, obstetric interventions including emergency cesarean occurred more frequently in the private cohort compared to women who opted for publicly funded care. The authors of this study concluded that the significant differences in obstetric intervention between the two groups were unlikely to be explained solely by differences in clinical risk factors alone. The results from our study suggest that a similar relationship may hold for neonatal outcomes as well.

The care women receive from experienced specialist obstetricians may play an important role in reducing adverse neonatal outcomes. There is evidence that private hospitals have shorter decision to intervention times, better communication regarding clinical care plans and are able to triage and treat more effectively in response to unanticipated clinical situations[26]. All these factors may result in better outcomes.

However, the data regarding the influence private health status on improved clinical outcomes is conflicting—a large study from the United States[27] showed that while private health insurance appeared to be protective against NCCU admission for some ethnic groups it was not for other racial groups. In this particular study having private insurance did not protect Black or Non-Hispanic mothers from higher neonatal intensive care admission rates than age-matched women with public insurance. The authors of this study suggest that adverse pregnancy outcomes were mitigated differently across ethnicity, maternal age and other factors and that possession of private insurance did not necessarily benefit all ethnic groups equally.

It is unclear if the differences in perinatal outcomes seen in our study are a direct result from differences in health care provision based on insurance status or whether the possession of private healthcare coverage was actually a proxy for other factors including the absence of poverty. Evidence suggests that people with private insurance are healthier and in less need of health care services than people with public insurance[28]. Privately insured women in our study had less likelihood of hypertension and gestational diabetes, two factors which are well known to influence perinatal outcomes.

A study from Western Australia[29] demonstrated that preterm infants of publicly funded women with similar demographics as privately insured women, had an increased risk of low Apgar score and took longer to establish unassisted respiration. However, they were less likely than infants of privately insured women to be admitted to a special care nursery for observation or treatment. The authors of this study made the point that, in Western Australia, there was a financial incentive for hospitals to admit babies to the neonatal unit whose mothers were privately insured because these infants then became separate fee paying patients. As a result of this dichotomy there might be an incentive to encourage admission of borderline infants for observation. The results from our study however do not support this hypothesis. One reason may be the fact that our study was confined only to term infants delivered by emergency cesarean. Another possibility is the fact that both public and private deliveries at the Mater Mothers’ Hospital take place in a single birth suite staffed by a common team of midwives. Obstetric and neonatal practice is also strictly governed by common institutional policies and guidelines. Our results are consistent with a previous Australian study of term births that found an increased risk of all adverse birth outcomes in the public cohort[15].

Another possibility to explain the disparities in neonatal outcomes seen in our study is the “weathering hypothesis”[30]. In our study there were greater numbers of indigenous and Asian women as well as those born outside of Australia in the public cohort. Given that Australia accepts a large number of migrants and refugees from some of the most socially disadvantaged regions of the world, the psychosocial stress resulting from their cumulative experiences may prematurely “age or weather” the reproductive system, contributing to the increased rates of poorer outcomes in the public cohort[30] [31]. Although it may be speculative at this stage, it is possible that factors influencing perinatal outcomes may go beyond ethnicity, insurance status, maternal co-morbidities and age to also include a woman’s previous social and psychological experiences.

The strengths of this study are its size and the fact that all deliveries took place within a single birth suite staffed by a common team of midwives within a large tertiary institution with comprehensive and contemporary policies and clinical guidelines. Availability of emergency operating theatre access and anesthetic staff were similar for both cohorts. Guidelines pertaining to admission to the neonatal unit applied equally to both the public and private cohorts. These uniformities minimized the potential for bias. We however also acknowledge the limitations of this study which relate mainly to its retrospective nature and potential for selection bias. We were also not able to ascertain the potential for differential misclassification of both the indication for the cesarean section or indeed the individual neonatal outcomes. Maternal lifestyle factors such as smoking was not always clearly recorded and therefore not analyzed. Smoking clearly influences perinatal outcomes and may be an important confounder which needs further evaluation. We also did not include differences of induction of labour rates between the two cohorts and this might have potentially influenced the outcomes. The influence of induction of labour on caesarean section rates and neonatal outcomes are however conflicting, with some studies [32] demonstrating no difference in either operative birth rates and others[33] suggesting an increase in intervention in the induction cohort. Other possible residual confounders including maternal educational level, number of antenatal visits, model of antenatal care, alcohol intake, or other barriers that may have influenced a woman’s experience in accessing care, were not measured.

### Conclusions

The results from this large study from a single tertiary centre suggests that perinatal outcomes after emergency caesarean section are better in women who had private obstetric care. However, caution is required in extrapolating our findings more generally. In particular, our findings need to be tempered by the fact that in many tertiary hospitals around the world, sicker and higher risk women are often cared for under the public system thereby leading one to expect that the disparity in health status alone could contribute to more adverse outcomes. The challenge for healthcare systems globally is to ensure that any inequalities are minimized between all cohorts of women.

## References

[1] Lipkind HS, Duzyj C, Rosenberg TJ, Funai EF, Chavkin W, Chiasson MA. (2009) Disparities in cesarean delivery rates and associated adverse neonatal outcomes in New York City hospitals. Obstet Gynecol 113: 1239–1247. doi: 10.1097/AOG.0b013e3181a4c3e5 19461418 10.1097/AOG.0b013e3181a4c3e5

[2] Einarsdottir K, Haggar F, Pereira G, Leonard H, de Klerk N, Stanley FJ, et al (2013) Role of public and private funding in the rising caesarean section rate: a cohort study. BMJ Open 3. 10.1136/bmjopen-2013-002789 PMC3646173 23645918

[3] Dahlen HG, Tracy S, Tracy M, Bisits A, Brown C, Thornton C. (2012) Rates of obstetric intervention among low-risk women giving birth in private and public hospitals in NSW: a population-based descriptive study. BMJ Open 2. 10.1136/bmjopen-2012-001723 PMC3467614 22964120

[4] Cai WW, Marks JS, Chen CH, Zhuang YX, Morris L, Harris JR. (1998) Increased cesarean section rates and emerging patterns of health insurance in Shanghai, China. Am J Public Health 88: 777–780. 9585744 10.2105/ajph.88.5.777 PMC1508965

[5] Lutomski JE, Murphy M, Devane D, Meaney S, Greene RA (2014) Private health care coverage and increased risk of obstetric intervention. BMC Pregnancy Childbirth 14: 13 doi: 10.1186/1471-2393-14-13 24418254 10.1186/1471-2393-14-13 PMC3898095

[6] Di Lallo D, Perucci CA, Bertollini R, Mallone S (1996) Cesarean section rates by type of maternity unit and level of obstetric care: an area-based study in central Italy. Prev Med 25: 178–185. 8860283 10.1006/pmed.1996.0044

[7] Coulm B, Le Ray C, Lelong N, Drewniak N, Zeitlin J, Blondel B. (2012) Obstetric interventions for low-risk pregnant women in France: do maternity unit characteristics make a difference? Birth 39: 183–191. doi: 10.1111/j.1523-536X.2012.00547.x 23281900 10.1111/j.1523-536X.2012.00547.x

[8] Al Rifai R (2014) Rising cesarean deliveries among apparently low-risk mothers at university teaching hospitals in Jordan: analysis of population survey data, 2002–2012. Glob Health Sci Pract 2: 195–209. doi: 10.9745/GHSP-D-14-00027 25276577 10.9745/GHSP-D-14-00027 PMC4168617

[9] Barros AJ, Santos IS, Matijasevich A, Domingues MR, Silveira M, Barros FC, et al (2011) Patterns of deliveries in a Brazilian birth cohort: almost universal cesarean sections for the better-off. Rev Saude Publica 45: 635–643. 21670862 10.1590/s0034-89102011005000039 PMC3794425

[10] Robson SJ, Tan WS, Adeyemi A, Dear KB (2009) Estimating the rate of cesarean section by maternal request: anonymous survey of obstetricians in Australia. Birth 36: 208–212. doi: 10.1111/j.1523-536X.2009.00331.x 19747267 10.1111/j.1523-536X.2009.00331.x

[11] Movsas TZ, Wells E, Mongoven A, Grigorescu V (2012) Does medical insurance type (private vs public) influence the physician's decision to perform Caesarean delivery? J Med Ethics 38: 470–473. doi: 10.1136/medethics-2011-100209 22562949 10.1136/medethics-2011-100209

[12] Mazzoni A, Althabe F, Liu NH, Bonotti AM, Gibbons L, Sanchez AL, et al (2011) Women's preference for caesarean section: a systematic review and meta-analysis of observational studies. BJOG 118: 391–399. doi: 10.1111/j.1471-0528.2010.02793.x 21134103 10.1111/j.1471-0528.2010.02793.x PMC3312015

[13] Zwecker P, Azoulay L, Abenhaim HA (2011) Effect of fear of litigation on obstetric care: a nationwide analysis on obstetric practice. Am J Perinatol 28: 277–284. doi: 10.1055/s-0030-1271213 21249618 10.1055/s-0030-1271213

[14] Roberts CL, Tracy S, Peat B (2000) Rates for obstetric intervention among private and public patients in Australia: population based descriptive study. BMJ 321: 137–141. 10894690 10.1136/bmj.321.7254.137 PMC27430

[15] Robson SJ, Laws P, Sullivan EA (2009) Adverse outcomes of labour in public and private hospitals in Australia: a population-based descriptive study. Med J Aust 190: 474–477. 19413516 10.5694/j.1326-5377.2009.tb02543.x

[16] Lucas DN, Yentis SM, Kinsella SM, Holdcroft A, May AE, Wee M, et al (2000) Urgency of caesarean section: a new classification. J R Soc Med 93: 346–350. doi: 10.1177/014107680009300703 10928020 10.1177/014107680009300703 PMC1298057

[17] ACOG (2009) Practice Bulletin No. 106—Intrapartum fetal heart rate monitoring: nomenclature, interpretation and general management principles. Obstet Gynecol: 192–202. 10.1097/AOG.0b013e3181aef106 19546798

[18] NICE (2007) CG 55. Intrapartum care: Care of healthy women and their babies during childbirth. 21250397

[19] RANZCOG (2014) Intrapartum Fetal Surveillance. Clinical Guideline—Third Edition 2014. (https://www.ranzcog.edu.au/intrapartum-fetal-surveillance-clinical-guidelines.html).

[20] Mhyre JM, Bateman BT, Leffert LR (2011) Influence of patient comorbidities on the risk of near-miss maternal morbidity or mortality. Anesthesiology 115: 963–972. doi: 10.1097/ALN.0b013e318233042d 21934482 10.1097/ALN.0b013e318233042d

[21] Fowler RA, Noyahr LA, Thornton JD, Pinto R, Kahn JM, Adhikari NK, et al (2010) An official American Thoracic Society systematic review: the association between health insurance status and access, care delivery, and outcomes for patients who are critically ill. Am J Respir Crit Care Med 181: 1003–1011. doi: 10.1164/rccm.200902-0281ST 20430926 10.1164/rccm.200902-0281ST PMC3269233

[22] Lyon SM, Benson NM, Cooke CR, Iwashyna TJ, Ratcliffe SJ, Kahn JM, et al (2011) The effect of insurance status on mortality and procedural use in critically ill patients. Am J Respir Crit Care Med 184: 809–815. doi: 10.1164/rccm.201101-0089OC 21700910 10.1164/rccm.201101-0089OC PMC3208649

[23] Vasquez DN, Das Neves AV, Aphalo VB, Loudet CI, Roberti J, Cicora F, et al (2014) Health insurance status and outcomes of critically ill obstetric patients: a prospective cohort study in Argentina. J Crit Care 29: 199–203. doi: 10.1016/j.jcrc.2013.11.010 24360595 10.1016/j.jcrc.2013.11.010

[24] Lombaard H, Pattinson RC (2008) Underlying medical conditions. Best Pract Res Clin Obstet Gynaecol 22: 847–864. doi: 10.1016/j.bpobgyn.2008.06.004 18675594 10.1016/j.bpobgyn.2008.06.004

[25] Karnad DR, Guntupalli KK (2004) Critical illness and pregnancy: review of a global problem. Crit Care Clin 20: 555–576, vii. doi: 10.1016/j.ccc.2004.05.001 15388189 10.1016/j.ccc.2004.05.001

[26] Olson R, Garite TJ, Fishman A, Andress IF (2012) Obstetrician/gynecologist hospitalists: can we improve safety and outcomes for patients and hospitals and improve lifestyle for physicians? Am J Obstet Gynecol 207: 81–86. doi: 10.1016/j.ajog.2012.06.045 22840717 10.1016/j.ajog.2012.06.045

[27] de Jongh BE, Locke R, Paul DA, Hoffman M (2012) The differential effects of maternal age, race/ethnicity and insurance on neonatal intensive care unit admission rates. BMC Pregnancy Childbirth 12: 97 doi: 10.1186/1471-2393-12-97 22985092 10.1186/1471-2393-12-97 PMC3495040

[28] Hindle D, McAuley I (2004) The effects of increased private health insurance: a review of the evidence. Aust Health Rev 28: 119–138. 15525264 10.1071/ah040119

[29] Einarsdottir K, Haggar FA, Langridge AT, Gunnell AS, Leonard H, Stanley FJ. (2013) Neonatal outcomes after preterm birth by mothers' health insurance status at birth: a retrospective cohort study. BMC Health Serv Res 13: 40 doi: 10.1186/1472-6963-13-40 23375105 10.1186/1472-6963-13-40 PMC3566968

[30] Geronimus AT (1992) The weathering hypothesis and the health of African-American women and infants: evidence and speculations. Ethn Dis 2: 207–221. 1467758

[31] Geronimus AT (2001) Understanding and eliminating racial inequalities in women's health in the United States: the role of the weathering conceptual framework. J Am Med Womens Assoc 56: 133–136, 149–150. 11759779

[32] Stock SJ, Ferguson E, Duffy A, Ford I, Chalmers J, Norman JE. (2012) Outcomes of elective induction of labour compared with expectant management: population based study. BMJ 344: e2838 doi: 10.1136/bmj.e2838 22577197 10.1136/bmj.e2838 PMC3349781

[33] Zhao Y, Flatley C, Kumar S (2017) Intrapartum intervention rates and perinatal outcomes following induction of labour compared to expectant management at term from an Australian perinatal centre. Aust N Z J Obstet Gynaecol 57: 40–48. doi: 10.1111/ajo.12576 28251626 10.1111/ajo.12576

